# Risk Factors for Rebleeding of Aneurysmal Subarachnoid Hemorrhage: A Meta-Analysis

**DOI:** 10.1371/journal.pone.0099536

**Published:** 2014-06-09

**Authors:** Chao Tang, Tian-Song Zhang, Liang-Fu Zhou

**Affiliations:** 1 Department of Neurosurgery, Huashan Hospital, Fudan University, Shanghai, China; 2 Department of TCM, Shanghai Jing-an District Central hospital, Shanghai, China; St Michael's Hospital, University of Toronto, Canada

## Abstract

**Background:**

Rebleeding is a serious complication of aneurysmal subarachnoid hemorrhaging. To date, there are conflicting data regarding the factors contributing to rebleeding and their significance.

**Methods:**

A systematic review of PubMed and Embase databases was conducted for studies pertaining to aneurysmal subarachnoid hemorrhage (aSAH) and rebleeding in order to assess the associated risk factors. Odds ratios (ORs) and corresponding 95% confidence intervals (CIs) were estimated from fourteen studies comprised of a total of 5693 patients that met the inclusion criteria.

**Results:**

Higher rebleeding rates were observed < 6 h after the initial aSAH (OR  = 3.22, 95% CI  = 1.46–7.12), and were associated with high systolic blood pressure (OR  = 1.93, 95% CI  = 1.31–2.83), poor Hunt-Hess grade (III–IV) (OR  = 3.43, 95% CI  = 2.33–5.05), intracerebral or intraventricular hematomas (OR  = 1.65, 95% CI  = 1.33–2.05), posterior circulation aneurysms (OR  = 2.15, 95% CI  = 1.32–3.49), and aneurysms >10 mm in size (OR  = 1.70, 95% CI  = 1.35–2.14).

**Conclusions:**

Aneurysmal rebleeding occurs more frequently within the first 6 hours after the initial aSAH. Risk factors associated with rebleeding include high systolic pressure, the presence of an intracerebral or intraventricular hematoma, poor Hunt-Hess grade (III-IV), aneurysms in the posterior circulation, and an aneurysm >10 mm in size.

## Introduction

Rebleeding is a major and disabling complication of aneurysmal subarachnoid hemorrhages (aSAH) with high mortality and morbidity [1]. It is therefore vital to identify contributing risk factors to enable early intervention and reduce the incidence of rebleeding. Factors associated with an increased risk for rebleeding include the time after initial aSAH [2]–[7], high systolic blood pressure [4], [5], [7], [8], low Glasgow coma scale score [9], poor Hunt-Hess grade [3]–[5], [7], [8], [10]–[13], intracerebral or intraventricular hematomas [3], [4], [5], [7]–[9], [13], [14], hemorrhage size [5], [10], [13], [15], hemorrhage location [3], [4], [5], [7], [13], [14], [16], number of aneurysms [14], presence of a sentinel headache [12], [14], early angiography [3], [10], [17], hyperglycemia [9], and levels of platelet sensitivity [3]. However, there are discrepancies regarding the significance of many of these predictors. As these factors have not been systematically examined, a systematic review of the scientific literature and quantitative meta-analyses were conducted to assess the associations of these factors with rebleeding.

## Methods

This study is in accordance with the Preferred Reporting Items for Systematic Reviews and Meta-Analyses (PRISMA) guidelines [18].

### Search strategy

A literature search of Pubmed and Embase databases was conducted for all studies (from the beginning of indexing to Dec 20, 2013) reporting risk factors for intracranial aneurysmal rebleeding. The search was restricted to English language articles and to clinical studies using the following key words: aneurysmal subarachnoid h(a)emorrhage, rebleeding, risk factors, and ruptured intracranial aneurysm. The references of included studies were also searched to identify relevant articles.

### Selection criteria

Database search results were initially screened by title or abstract, and the identified relevant studies were reviewed in detail. The criteria for inclusion of relevant studies were as follows: 1) reports of confirmed aSAH cases; 2) studies allowing for the extraction of odds ratios (OR) with the corresponding 95% confidence intervals (CI); 3) at least three articles referring to a given risk factor; 4) cohort or case-control studies or randomized controlled studies. Studies on animals, systematic reviews and individual case reports were excluded.

### Data extraction

Two authors (CT and TSZ) independently assessed the eligibility of all studies and disagreements were resolved by discussion or in consultation with the third author. The following data were extracted from eligible studies: author, year of publication, country, rebleed number, sex, and mean age. Rebleeding was diagnosed based on the following: 1) symptoms of aSAH: severe headache, a decrease in the level of consciousness, rapid changes in vital signs, *etc.*; 2) previously confirmed aSAH; 3) increased volume of blood measured from computed tomography (CT) scan as compared to a previously recorded CT.

### Quality analysis

The quality of the included studies was independently assessed by two authors using the Newcastle-Ottawa Scale (NOS) [19]. A total score of 0 to 9 points was assigned to each study determined by a maximum of 4 points given for patient selection, 2 points for comparability of groups, and 3 points for outcome report.

### Data analysis

Stata version 12.0 (Stata Corp LP; College Station, Texas, USA) was used for data analyses. For comparison of rebleeding results, the OR was used as the effect indicator. The OR and 95% CI values were pooled directly from the study reports, or were obtained from the available data. ORs and 95% CIs were estimated using the Mantel and Haenszel method. Heterogeneity among studies was assessed using I^2^-statistics (I^2^ = 0–25%, no heterogeneity; I^2^ = 25–50%, moderate heterogeneity; I^2^ = 50–75%, large heterogeneity; I^2^ = 75–100%, extreme heterogeneity) [20], [21]. The random effects model was used when heterogeneity was indicated, and the fixed effects model was used in cases of no heterogeneity. The robustness of the combined results was confirmed in studies with large or extreme heterogeneity using the “leave one out” sensitivity analysis [22]. In addition to the comparison among all subjects, a stratification analysis of systolic blood pressure cut-off points was performed. The Begg's funnel plot and Egger's test for possible publication bias were not used as there were less than ten studies included in each risk factor category.

## Results

### Study characteristics

**Figure 1:**
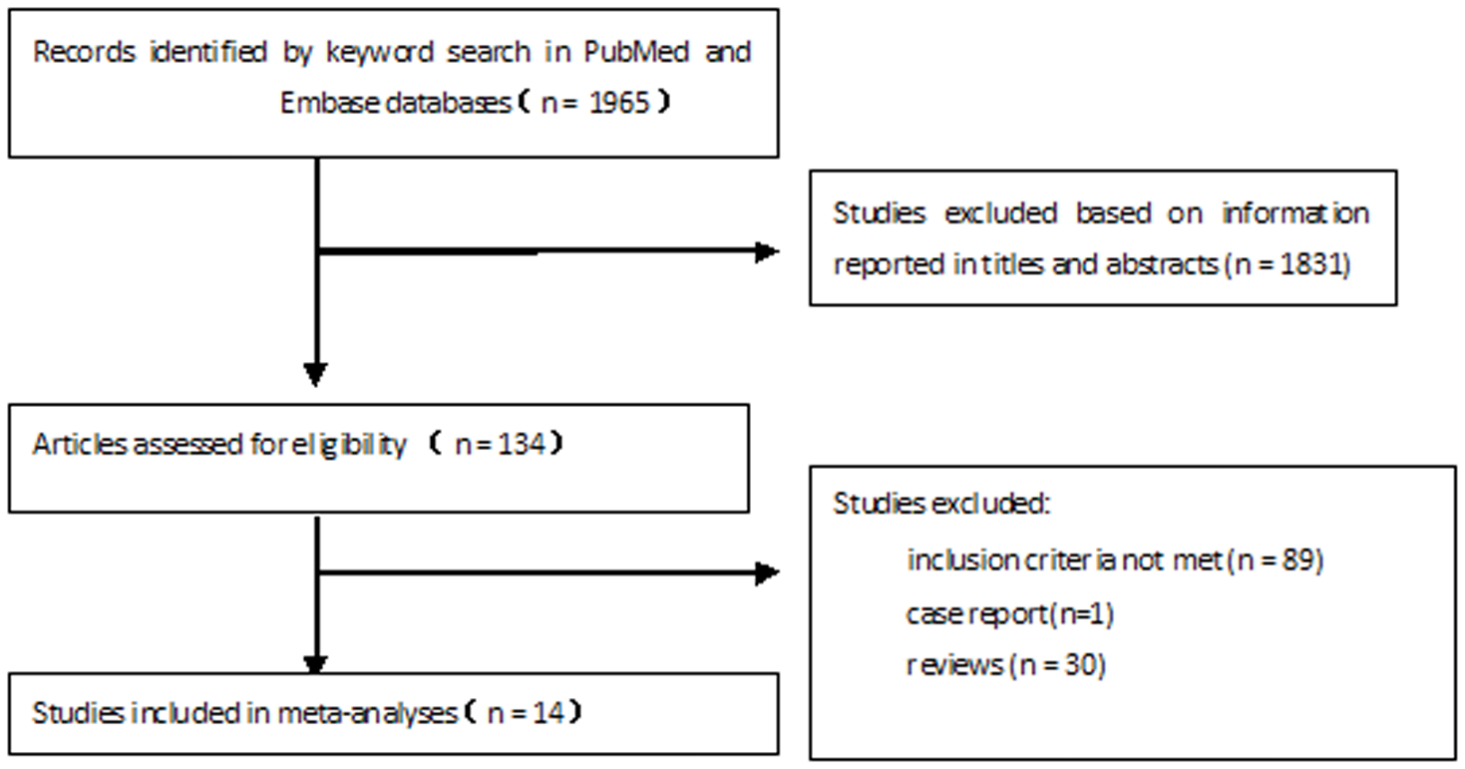
Flowchart describing the selection of studies included in the meta-analyses.

An initial search of the electronic databases yielded 1965 articles, of which 134 were selected as relevant based on their title and abstract. Fourteen of these met the inclusion criteria (Fig. 1) and were used for analyses of six identified risk factors: time after initial aSAH, systolic blood pressure, Hunt-Hess grade, intracerebral or intraventricular hematoma, size of hemorrhage and location of aneurysm. These studies were of moderate or high quality (NOS score >5) and included a combined total number of 5693 case subjects from populations within the United States, China, Germany, the Netherlands, Finland, Japan and Korea. The subject sample sizes in the included studies ranged from 179 to 1312. The baseline characteristics of each included study are presented in Table 1.

**Table 1:** Characteristics of included studies.

Author	Country	Year	Sample size	mean age	M/F	rebleeding	Quality
Inagawa T	Japan	1987	150	—	—	33	5
Ando T	Japan	1989	661	—	—	65	5
Juvela S	Finland	1989	236	—	—	53	5
Aoyagi N	Japan	1996	239	—	—	62	5
Fujii Y	Japan	1996	179	—	—	31	7
Obkuma H	Japan	2001	273	58.6±12.3	90/183	37	6
Naidech AM	United States	2005	574	53.1±15	190/384	40	6
Beck J	Germany	2006	237	53.4±13.9	98/139	23	6
Machiec P C	Netherlands	2006	354	—	—	90	7
Kitsuta Y	Japan	2006	202	58.0±13.0	76/126	42	5
Cha KC	Korea	2010	492	—	182/310	38	6
Guo LM	China	2011	326	—	130/196	70	7
Cong W	China	2012	458	—	179/279	63	7
De Marchis GM	United States	2014	1312	—	435/877	113	7

### Time interval

**Figure 2:**
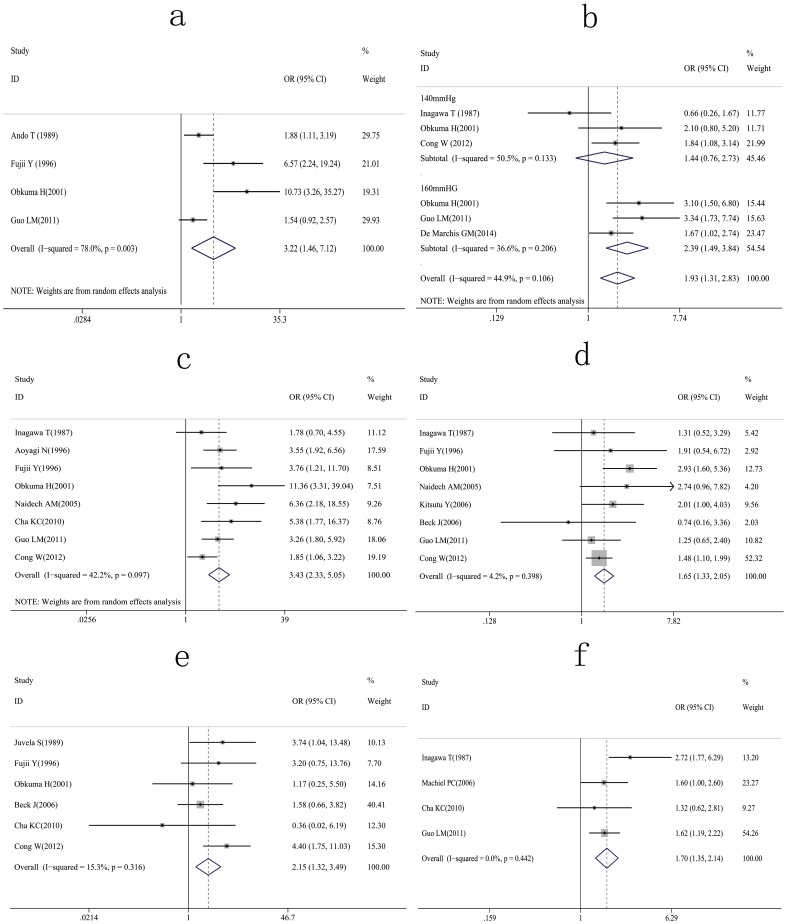
Forest plots for the relation between rebleeding and (a) interval time (<6 h) after initial aSAH; (b) systolic blood pressure; (c) Hunt-Hess grade; (d) intracerebral or intraventricular hematomas; (e) location of aneurysms (posterior *vs.* anterior circulation); (f) aneurysm size (>10 mm).

The time intervals after aSAH were reported as <6 h [2]–[5], <24 h [6], [23], and <48 h [7]. Analyses showed that a significantly increased risk of rebleeding was associated with a time interval <6 h after the initial aSAH (Fig. 2a). In one of the four studies reporting rebleeding <6 h after the first aSAH, the time interval between the last attack and admission was a strong independent predictor, but the OR was not provided [3]. The OR was based on the number of rebleeding events in each time interval and calculated as the odds of rebleeding <6 h after aSAH/odds of rebleeding >6 h after aSAH. A significantly increased rate for rebleeding was found <6 h after initial aSAH (OR  = 3.22, 95% CI  = 1.46–7.12). Extreme heterogeneity among these studies was indicated (I^2^  = 78.0%; *P*  = 0.003), and a sensitivity analysis revealed that the OR value was not significantly changed even with the exclusion of a single study (Table 2).

**Table 2:** Sensitivity analysis of studies evaluating the time interval (<6 h) for rebleeding.

study (year)	OR	95% Conf. Interval	
Ando T (1989)	4.41	1.15	16.84
Fujii Y (1996)	2.62	1.14	6.01
Obkuma H(2001)	2.27	1.20	4.33
Guo LM (2011)	4.66	1.44	15.12
Combined	3.22	1.46	7.12

### Blood pressure

Systolic blood pressure was investigated in five studies [4], [5], [7], [8], [24], with multivariate analyses in three of these studies [5], [7], [24]. For studies that did not provide OR directly, the OR was calculated as the odds of rebleeding with high systolic pressures/odds of rebleeding with control pressures. For these analyses, a high blood pressure was defined as greater than either 140 mmHg or 160 mmHg. Of the three studies using 140 mmHg to delineate high pressure [4], [7], [8], two studies found no significant association between rebleeding and systolic pressure [4], [7], and the third listed the number of rebleeding events in three categories (<139 mmHg, 140–179 mmHg, >180 mmHg) [8]. Three studies found a significant association with high systolic pressure (>160 mmHg) and rebleeding [4], [5], [24]. A meta-analysis of these studies revealed that a systolic blood pressure >160 mmHg is more closely associated with rebleeding (OR  = 2.39, 95% CI  = 1.49–3.84) than a systolic blood pressure >140 mmHg (OR  = 1. 44, 95% CI  = 0.76–2.73) (Fig. 2b).

### Hunt-Hess grade

Eight studies investigating the relationship between rebleeding and Hunt-Hess grades were identified [3]–[5], [7], [8], [11]–[13], and multiple logistic analyses were used in four of these studies [3], [5], [7], [12]. Of these four, one study showed a negative association between Hunt-Hess grades III–IV and rebleeding [7], another study found no association [3], and two studies reported the Hunt-Hess grade as an independent risk factor [5], [12]. These studies listed the number of patients with rebleeding in each Hunt-Hess category, and results were pooled into either a good condition (grade I–II), or a poor condition (grade III–IV) group. The results indicate a relationship between rebleeding and a poor condition (OR  = 3.43, 95% CI  = 2.33–5.05) (Fig. 2c).

### Hematomas

The relationship between intracerebral or intraventricular hematomas and rebleeding were reported in eight studies [3]–[5], [7]–[9], [12], [14]. One logistic regression analysis showed that intracerebral or intraventricular hematomas were independent risk factors [7], while another study suggested they were a risk factor for ultra-early rebleeding, but not independent predictors of rebleeding [3]. The remaining six studies listed the number of rebleeding incidents in patients with intracerebral or intraventricular hematomas and the OR was calculated as the odds of rebleeding in intracerebral or intraventricular hematomas/odds of rebleeding in the remaining aSAH cases. The results indicate a relationship between rebleeding and intracerebral or intraventricular hematomas (OR  = 1.65, 95% CI  = 1.33–2.05) (Fig. 2d).

### Aneurysm location

Six studies examined the relationship between the location of aneurysms and rebleeding [3]–[5], [7], [13], [14], [16], with univariate analyses in two of these revealing a higher rebleeding rate in posterior circulation aneurysms [7], [14]. For these studies, the number of rebleeds from posterior and anterior circulation aneurysms was counted and the OR was calculated as the odds of rebleeding in posterior circulation aneurysms/odds of rebleeding in anterior circulation aneurysms. The results of the meta-analysis indicate that there is a significantly increased risk of rebleeding with posterior circulation aneurysms (OR  = 2.15, 95% CI  = 1.32–3.49) (Fig. 2e).

### Aneurysm size

Four studies investigated the relationship between aneurysm size and risk of rebleeding [5], [8], [13], [15], with multivariate analyses used in two studies [5], [15]. One study reported size as an independent risk factor [5], while another study estimated crude and age-adjusted hazard ratios of aneurysm size [15]. The remaining two included studies listed the number of rebleeds occurring in patients with aneurysms >10 mm [8], [13]. The OR was calculated as the odds of rebleeding in patients with aneurysms >10 mm/odds of rebleeding in patients with aneurysms <10 mm. Results of this analysis confirm that an aneurysm >10 mm in size is a risk factor for rebleeding (OR  = 1.70, 95% CI  = 1.35–2.14) (Fig. 2f).

## Discussion

Rebleeding occurs most frequently in the early stages following an aSAH (within the first 2–6 h), known as ultra-early rebleeding [2]–[4], [7], [8]. The present study documents substantial heterogeneity in studies reporting rebleeding rates within the first 6 h after the initial aSAH. This heterogeneity likely reflects the fact that observations were made in various settings, either outside of a hospital [4], in-hospital [3], or not indicated [2], [5]. While a sensitivity analysis showed a moderate difference, the pooled estimate of rebleeding rates did not vary substantially with the exclusion of any one study. It was suggested that the early risk for rebleeding is a consequence of activated fibrinolysis and reduced clot-stability during the first 6 h [25]. Accordingly, early anti-fibrinolytic treatments can be effective for the prevention of rebleeding [26], [27].

Many studies have reported that elevated systolic pressure after the first aSAH leads to a higher risk for rebleeding [4], [5], [7]. It is thought that the elevated systolic pressure might increase the transmural pressure beyond the compliance of the hemostatic clot and result in rebleeding. Different cut-off points have been used throughout the literature to define high systolic pressure, ranging from 140–180 mmHg. Our subgroup analysis showed that systolic pressure fails to predict rebleeding when 140 mmHg was the delineative pressure value. However, when a threshold value of 160 mmHg was employed, high systolic pressure was more predictive of rebleeding. The results of our meta-analysis therefore suggest that high systolic blood pressure can be associated with aneurysm rebleeding, though this increase in risk was not significant in all of the assessed studies [3], [6]. A possible explanation is that pharmacologic interventions to lower systolic pressure are usually routinely performed in these cases [4]. Accordingly, significantly higher rebleeding rates were more commonly found in patients with elevated systolic pressure in the very early stage during transport [28]. There is general agreement that blood pressure control in acute aSAH is essential for the prevention of rebleeding. However, the magnitude of blood pressure control has not been established, though some studies report maintenance of pressures below 140 mmHg [17], [28]. The results of this study indicate that pressures need only be maintained below 160 mmHg to reduce the risk of rebleeding. One concern for blood pressure control is whether it may lead to cerebral ischemia. However, a prospective case-controlled study has shown that systolic pressure <160 mmHg has no adverse effect on regional brain tissue oxygen levels when intravenous nicardipine is used for the treatment of hypertensive neurologic emergencies [29].

In the included studies, the patient's clinical status was assessed using the Hunt-Hess scale. Several investigators have reported that a poor Hunt-Hess grade is significantly related to aneurysmal rebleeding [3]–[5], [7], [8], [11]–[13]. The results of this meta-analysis support these findings, as a higher Hunt-Hess grade was associated with a greater incidence of rebleeding. One study suggested that rebleeding rates in patients with grade V were lower compared to patients with grade III and grade IV [8]. The reason for this discrepancy might be that detection of rebleeding may be missed on CT scans or by clinical signs, due to the resultant coma state. To eliminate this potential bias, the data of grade V was not included in the present study.

After the first rupture of an aneurysm, the distribution and amount of the initial hemorrhage could affect the mortality, and contribute to delayed arterial vasospasm [1], though no relationship has been found between rebleeding and the volume of the subarachnoid clot [3], [30]. Intracerebral or intraventricular hematomas were identified as risk factors for rebleeding by Cong and his colleagues [7]. Although this association was confirmed, further study is needed, as no increased risk was observed when this analysis was restricted to subjects within 6 h after aSAH. Thus, the rebleeding rate may be affected by the timing of the observation [8]. A variety of other parameters associated with intracerebral or intraventricular hematomas could also affect the rebleeding rate, such as poor clinical grade, high blood pressure, acute hydrocephalus and following external ventricular drainage [12], though these factors have not been adequately explored in the majority of these studies.

Rebleeding rates were compared between aneurysms within the anterior and posterior circulations. Results indicate that anterior circulation aneurysms are associated with a lower rate of rebleeding, consistent with previous studies [7], [14]. Two previous studies have shown that patients with larger aneurysms have an increased risk for rebleeding [5], [13], [15], with adjusted-age as a potential confounding factor [15]. This analysis confirms this association, identifying aneurysms >10 mm in size as a risk factor for rebleeding.

### Limitations

To our knowledge, this analysis provides the first comprehensive evidence-based assessment of relevant factors for rebleeding. However, several potential limitations of our analyses should be considered. First, most of the included studies were from Asia,thus the findings may not universally represent all cases. Second, the collected data originated from case-controlled studies as a prospective study cohort was unavailable, making it difficult to establish the timeline of rebleeding risk factors. Additionally, the calculations of ORs by multivariate or univariate analyses were not restricted in this study. The presence of conflicting data on the predictors for rebleeding reflects the variations in study design, treatment strategy, definition of study variables, and the observation time, all of which may influence the results and lead to bias. To perform the meta-analysis, continuous covariates were dichotomized. For example, in systolic pressure analysis, there is no generally accepted cut-off point for defining high systolic pressure to predict rebleeding. In fact, dichotomization could lead to serious bias [31]. Therefore, to minimize the bias, cut-off points of systolic pressure or Hunt-Hess grade were made according to the clinical justification.

## Conclusions

In conclusion, this study indicates that aneurysmal rebleeding occurs more frequently within 6 h after the initial aSAH, with elevated systolic pressures, poor Hunt-Hess grades, intracerebral or intraventricular hematomas, aneurysms >10 mm in size, and aneurysms in the posterior circulation. The limitations of the included studies stress the need for prospective, large, observational studies with clearly defined methodologies, and sufficient sample sizes to assess independent prognostic risk factors for rebleeding.

## Supporting Information

Checklist S1 PRIMSA checklist of this meta-analysis.
(DOC)
